# A Novel Artificial-Intelligence-Based Reverberation-Reduction Algorithm for Cochlear Implants Enhances Speech Intelligibility and User Experience

**DOI:** 10.1097/AUD.0000000000001745

**Published:** 2025-11-03

**Authors:** Nienke C. Langerak, H. Christiaan Stronks, Esther F. van Marrewijk, Jeroen J. Briaire, Jean-Marie Lemercier, Timo Gerkmann, Johan H.M. Frijns

**Affiliations:** 1 Department of Otorhinolaryngology and Head & Neck Surgery, Leiden University Medical Center, Leiden, the Netherlands; 2 Leiden Institute for Brain and Cognition, Leiden University, Leiden, the Netherlands; 3 Signal Processing, Universität Hamburg, Hamburg, Germany; 4 Department of Bioelectronics, Delft University of Technology, Delft, the Netherlands.

**Keywords:** Artificial intelligence, Cochlear implant, Preprocessing, Reverberation, Sensorineural hearing loss

## Abstract

**Objectives:**

Cochlear implantation is the standard of care for severe-to-profound hearing loss in the Netherlands. Cochlear implants (CIs) generally perform well in quiet conditions, but speech understanding in an environment with reverberations remains difficult. This study aimed to minimize the amount of reverberation present in a speech signal by using novel artificial-intelligence-based algorithms created for use in CIs.

**Design:**

A prospective crossover study was performed, which included 15 CI users, with each participant being their own control. Two versions of the algorithm were tested: one version that focused on late reverberations (DNN-WPE) and another version that additionally minimized early reverberation with a post-filter (DNN-WPEPF). These two algorithms were tested by performing speech intelligibility tests with percentage correct as the outcome measure. The Flemish/Dutch Matrix test was used for speech intelligibility testing. Six different conditions were measured: clean speech (no reverberation), clean speech processed with both algorithms, reverberated speech, and reverberated speech processed by both algorithms. In addition, subjective ratings were performed to assess how the participant perceived the processed sound. These subjective ratings were performed by pairwise comparisons of the aforementioned conditions regarding listening effort, naturalness, and speech intelligibility.

**Results:**

The speech intelligibility scores revealed a statistically significant average improvement of 11% when reverberated speech was processed with DNN-WPE (*p* < 0.001) and 17% when processed with DNN-WPEPF (*p* < 0.001). Moreover, the benefit of DNN-WPEPF was significantly greater than the benefit of DNN-WPE (*p* = 0.018). Both algorithms did not significantly affect speech intelligibility when no reverberation was present (*p* > 0.05). The outcomes of the three subjective ratings complement the speech intelligibility scores. Speech dereverberated with either algorithm was significantly preferred over reverberated speech for all three outcomes (listening effort, naturalness, and speech intelligibility). Moreover, speech dereverberated with DNN-WPEPF was significantly preferred over speech dereverberated with DNN-WPE.

**Conclusions:**

This study revealed that the DNN-WPE and DNN-WPEPF dereverberation algorithms had benefits for CI users regarding speech intelligibility and subjective ratings. These algorithms did not affect the clean speech, showing that they can be switched on in quiet situations without background noise. Further developments are required to implement the algorithms in real time on the CI processor, and more research is needed to assess them under more realistic listening conditions.

## INTRODUCTION

Cochlear implantation is the standard of care for severe-to-profound hearing loss in the Netherlands. Cochlear implants (CIs) often perform well under quiet conditions, with some users achieving 100% speech understanding; however, speech understanding in the presence of background noise remains difficult for most CI users (Hu & Kokkinakis 2014; Langerak et al. 2024). Background noise can include stationary and fluctuating noise, with fluctuating noise being more detrimental to speech understanding among CI users (Stronks et al. 2020). Another commonly encountered type of fluctuating interference that is detrimental for speech understanding in CI users is reverberation (Hazrati & Loizou 2012; Hu & Kokkinakis 2014).

Reverberations are time-delayed reflections of an original sound signal, which cause temporal smearing and blurring of spectral cues of the original sound (Assmann et al. 2004; Hazrati & Loizou 2012). Reverberation includes both early and late reflections (Hu & Kokkinakis 2014). The early reflections occur within 50 to 100 msec, and are perceptually integrated with the direct sound, effectively making the direct sound louder. For typical-hearing listeners and for hearing-impaired hearing aid (HA) users, early reflections provide the benefit of increasing speech intelligibility (Bradley et al. 2003; Habets 2007). For CI users, there is debate about the effect of early reverberations on speech intelligibility. Hu and Kokkinakis (2014) state that early reflections are not helpful, but rather degrade speech intelligibility for CI users. By contrast, the study of Kressner et al. (2018) revealed that early reflections are not detrimental for CI recipients. They concluded that the presence of late reflections in combination with the source-receiver distance in moderately to highly reverberant rooms resulted in a detrimental effect on speech intelligibility for CI recipients. These results were confirmed by the study of Eurich et al. (2019), who also investigated speech pleasantness scores for different environments. Despite the debate on the effects of early reflections on speech intelligibility, there is a consensus on the detrimental effect of late reflections.

Late reflections occur after 100 msec, and cannot be integrated with the direct sound. Thus, late reflections are perceived as reverberation and are detrimental for typical-hearing listeners, HA users, and CI users. Still, CI users experience the most profound effects on speech intelligibility. This is due to the way CI speech processors convert sound, namely through envelope coding, in which slow modulations (i.e., the envelope) are transmitted to the CI, while the temporal fine structure is typically discarded. Reverberation alters the envelope by decreasing the amplitude modulations (temporal smearing) (Assmann et al. 2004). Because CI users rely on this envelope, they experience reduced speech understanding.

The magnitude of reverberation is determined by the characteristics of the room, and primarily by its dimensions and the material of the walls. One of the parameters to describe the magnitude is the reverberation time (RT60), which describes the time from when the original sound emission stops until the sound signal is reduced by 60 dB SPL (Zuckerwar 2003). A higher RT60 is associated with greater difficulty understanding speech (Kokkinakis et al. 2011). Another measure to express the magnitude of reverberation is the direct-to-reverberant ratio (DRR) and the source-to-listener distance. The DRR expresses the ratio of the sound pressure level of the direct sound and the sound pressure level of the reverberant sound. The more positive the DRR or the smaller the source-to-listener distance, the higher the speech intelligibility and vice versa (Badajoz-Davila et al. 2020).

Reducing the reverberation (dereverberation) of a signal can substantially increase speech intelligibility for CI users. HAs use many dereverberation approaches based on classical signal processing, including spectral enhancement, beamforming, coherence weighting, and methods based on linear prediction, such as the weighted-prediction error (WPE) algorithm (Lebart et al. 2001; Gerkmann 2011; Naylor & Gaubitch 2011; Cauchi et al. 2014; Jukić et al. 2015). To date, few approaches have been developed specifically for CIs, without requiring a priori knowledge of the reverberant environments and the use of restricted computational power (Kokkinakis et al. 2011; Hazrati & Loizou 2013; Hazrati et al. 2013; Shahidi et al. 2022). Gaultier and Goehring (2024) showed that a deep-learning algorithm was able to improve speech intelligibility in noisy-reverberant situations for CI users. The signal processing group of the University of Hamburg has designed a new algorithm to allow CIs to minimize the reverberation of a speech signal (Lemercier et al. 2022, 2023). This new technique is based on a deep neural network (DNN) and the WPE method (DNN-WPE). The WPE algorithm models the reverberation process as an autoregressive model, in which reverberation at a given time can be predicted from previous samples of reverberant microphone signals. Thus, the desired speech signal can be estimated as the prediction error (Jukić et al. 2015). Speech signal estimation requires the power spectral density (PSD) of the non-reverberant signal. A DNN was trained to model the PSD of non-reverberant speech material, which then serves as input for the WPE algorithm to minimize reverberation (Lemercier et al. 2022, 2023). The DNN-WPE-based algorithm was initially developed to minimize late reflections. However, because CI users can experience reduced speech intelligibility due to both early and late reflections, the parameters of DNN-WPE have been proposed to be adjusted to remove early reflections when used in CIs. In addition, for longer reverberation times, modeling the inverse filter via WPE is challenging and computationally demanding. Thus, to also efficiently remove the tail of late reflections, Lemercier et al. (2023) proposed to combine the tailored DNN-WPE algorithm with a post-filter (PF) designed to cancel the remaining late reverberations (DNN-WPEPF). Additional details are available in the publications of Lemercier et al. (2022) and Lemercier et al. (2023). Both algorithms were trained on reverberation times from 0.4 to 1.0 sec, which correspond to environments commonly encountered in daily life (office space, living room, classroom, etc.). The WHAMR dataset, which is based on American English speech of the Wall Street Journal, was used as reverberant training material, modeled in rooms of different dimensions (Garofolo et al. 1993; Maciejewski et al. 2020). In a scenario simulating conditions of CI users listening to speech in reverberation, instrumental metrics suggest significant improvements in speech quality and dereverberation performance for both algorithms, with an even larger improvement with the PF (Fig. 3 in Lemercier et al. 2023).

In the present study, the aim was to analyze the effectiveness of the DNN-WPE algorithm, with and without PF, in a clinical setting with unilateral CI users. This work builds upon the previous acoustic analysis metrics of Lemercier et al. (2022, 2023) by extending the evaluation to human subjects. The effectiveness of this algorithm was assessed using speech intelligibility tests and by determining the study participants’ subjective perception of the dereverberated speech. To evaluate subjective perception, participants were exposed to pairs of listening conditions, with versus without dereverberation, and rated their preferred condition in terms of speech intelligibility, listening effort, and naturalness.

## MATERIALS AND METHODS

### Study Design and Participants

A prospective study including 15 CI users was performed, with each subject as their own control. Testing was performed acutely using each participant’s standard clinical program as well as both dereverberation algorithms. Only the experimenter was aware of the condition being tested. All participants had a unilateral CI, and had at least 6 months of experience with their CI. Additional inclusion criteria were fluency in Dutch, age of at least 18 years, and a consonant-vowel-consonant phoneme score in quiet of at least 75% at 65 dB SPL with only their CI. Participants were excluded if they had disorders other than hearing impairment, which could impact their testing ability. Table 1 presents the demographics of the CI users. This study was approved by the Medical Ethics Committee Leiden, The Hague, Delft (METC protocol number NL67179.058.18).

**TABLE 1.** Participants’ demographics

Subject ID	Sex	Age (yrs)	CVC Score (%)	CI Experience (mos)	CI Processor	CI Side	Etiology	RT60_50%_ (s)
DEV02	F	71	91	172	Q90	AS	Familial	1.1
DEV03	M	77	83	148	M90	AS	Ménière disease	1.2
DEV04	F	71	86	94	M90	AD	Unknown	1.2
DEV05	M	68	93	269	M90	AS	Congenital	1
DEV06	F	61	93	276	Q90	AS	Unknown	1.45
DEV07	M	71	89	116	Q90	AD	Otosclerosis	0.8
DEV08	M	76	86	29	M90	AD	Familial	0.6
DEV09	M	75	88	53	Q90	AS	Familial	0.6
DEV10	F	68	76	56	Q90	AD	Congenital	1.1
DEV11	M	79	85	173	M90	AS	Ménière disease	0.8
DEV13	M	77	86	11	M90	AS	Congenital	1
DEV14	M	79	94	199	M90	AD	Otosclerosis	1
DEV15	F	56	93	34	M90	AD	Meningitis	1.3
DEV16	M	49	90	12	M90	AD	Unknown	1.1
DEV17	M	67	86	14	M90	AS	Familial	0.85
Median (min–max)		71 (49–79)	88 (76–94)	94 (11–276)				1.0 (0.6–1.45)

CI, cochlear implant; CVC, consonant-vowel-consonant phoneme score; F, female; ID, identification number; M, male; M90, Marvel 90 Advanced Bionics processor; Q90, Naida CI Q90 Advanced Bionics processor; RT60_50%_, the reverberation time for which a participant understood 50% of the sentences correctly.

### Measurement Setup and Calibration

Study participants received a research Marvel™ 90 (M90) CI processor (Advanced Bionics LLC, Valencia, CA, USA) fitted with their clinical program, including M and T levels. If a participant had a Q90 processor (a previous generation of the M90), their clinical settings were transferred to the clinical fitting software for the M90 processor, using Target CI^TM^ (version 1.0.20; Advanced Bionics LLC, Valencia, CA, USA) via the SoundWave^TM^ Client Data Migration option. For testing, we used direct Bluetooth streaming to the processor. During streaming, the microphones on the CI processor were turned off to prevent interference from noises and sounds in the room. Clinical preprocessing algorithms (e.g., Soundrelax^TM^ and WindBlock^TM^) were turned off. Participants removed any HAs, and their non-implanted ear was plugged.

To calibrate the Bluetooth signal, we recorded the voltage output of the M90 processor using a digital oscilloscope (LabNation Smartscope BVBA, Antwerp, Belgium) connected to the processor by the M Listening Check^TM^ (Advanced Bionics LLC, Valencia, CA, USA). For this purpose, a 1-kHz sine wave of 60 dBA was presented in the free field over a loudspeaker (KEF, Ci100QS; GP Acoustics, Kent, United Kingdom) that had been calibrated using a sound level meter (RION NL-52; Sysmex, Etten-Leur, the Netherlands) with a resolution of 0.1 dB SPL. The corresponding voltage output of the M90 CI speech processor was compared with that of the M90 CI speech processor when presented with the same 1-kHz sine wave via Bluetooth streaming. The voltage output of the M90 CI speech processor in both situations (1-kHz played in free field and 1-kHz played via Bluetooth) was equated by adjusting the volume of the laptop’s system sound (resolution <0.1 dB SPL) to create a loudness of 60 dBA when presenting a fragment via Bluetooth. Subsequently, the volume of the laptop’s system sound was kept constant.

### Dutch/Flemish Matrix Sentence Test

To evaluate the impact of the DNN-WPE and DNN-WPEPF algorithms on reverberated speech, the Dutch/Flemish Matrix test was used (Luts et al. 2014). This speech corpus consists of 13 lists, each containing 20 sentences comprising 5 short words: a name, verb, number, color, and an object. The Matrix sentences were incorporated into a custom-built speech-in-noise test in a MATLAB R2023a environment. Participants had no visual representation of the Matrix sentences and verbally repeated the words they recognized; guessing was allowed. The response was manually scored by the experimenter, and no feedback was given to the participants.

### Signal Processing

To generate reverberant speech material with varying amounts of reverberation, different RT60s were introduced into the Matrix sentences. To introduce reverberation, as in Lemercier et al. (2022, 2023), a room impulse response simulation was conducted, using a virtual room with length × width × height measurements of 7 × 6 × 3.5 m (Wendt et al. 2014). In this virtual room, a head was modeled (radius of 16 cm) with a distance to the source of 1.9 meters. Two different signals were generated: one for the left ear and one for the right ear. We chose to use the signal of only one ear, because our study participants listened monoaurally. The RT60 ranged from 0.4 to 1.5 sec, with intervals of 0.05 sec. The room had the same dimensions and source-to-listener distance for every RT60. The RT60 was modeled by adjusting the reflection coefficient of the surfaces in the room model. All surfaces, including the floor and the ceiling, were modeled with the same properties. Scattering and directionality of sounds were not taken into account. The DRR of the simulation was ‐3.4 for an RT60 of 0.4 sec and ‐8.5 for an RT60 of 1 sec. Following the addition of signal reverberation, the sentences were dereverberated with the DNN-WPE and DNN-WPEPF algorithms.

Previous research using computer models has shown that the algorithms work optimally after a 4-sec initialization period (Lemercier et al. 2022). In practice, the filter is guaranteed to converge to a stable value already after up to 2 sec (the initialization duration of 4 sec was chosen due to training data formatting reasons), yielding the optimal dereverberation performance (unpublished observations). In our present study, the sentences of the matrix test were each approximately 1.8 sec long. Thus, to create an initialization period, we concatenated each individual sentence five times. After these concatenated sentences were processed with the algorithms using Python (version 3.10), the fourth sentence was extracted for presentation to the participant. This protocol allowed sufficient time for the algorithm to initialize and eliminated any termination artifacts. Because only the fourth sentence was presented to the participant, no learning effect can have occurred from the repeated presentation of each sentence. Also, the learning effect for the algorithms was expected to be minimal because most of the neural network modeling capacity was allocated to the actual task of anechoic PSD estimation from reverberant speech, which only requires a few hundred milliseconds (modeling of the reverberation tail and speech characteristics). The absence of sentence learning by the algorithm was confirmed by a test with a few participants, where we initialized the algorithm with different sentences in front of the fourth sentence we presented to the participant. This resulted in the same benefits of the algorithms as the repetition of the same sentence, which indicates that the neural network did not use the initialization sentences to generate its output, but that the sentences merely served to initialize the WPE filter.

### Speech Intelligibility Testing

Each participant underwent one session of approximately 2.5 h. At the start of the session, the RT60 at which speech intelligibility was 50% (RT60_50%_) was determined by testing a range of unprocessed RT60s (0.4 to 1.4 sec), subsequently followed by a linear interpolation. For each RT60, 10 sentences from the Dutch/Flemish matrix test were presented at 60 dB SPL. The 50% threshold for the reverberated condition was determined to assess the effect of both algorithms at the steepest point of the curve. This approach allowed us to observe the optimal effectiveness of the algorithms. The RT60_50%_ was determined for each individual at the beginning of the session and was held constant for the remainder of the session. Subsequently, speech-in-noise tests were conducted under six different conditions: clean speech (no reverberation or algorithm), clean speech with DNN-WPE algorithm, clean speech with DNN-WPEPF algorithm, reverberated speech, reverberated speech with DNN-WPE algorithm, and reverberated speech with DNN-WPEPF algorithm. The conditions with reverberation were tested with the participants’ previously determined RT60_50%_. For each of the six measurement conditions, a test and a retest were performed. The testing order was randomized, and thus differed for all participants, including the test and retest.

### Subjective Ratings

**Fig. 1.**
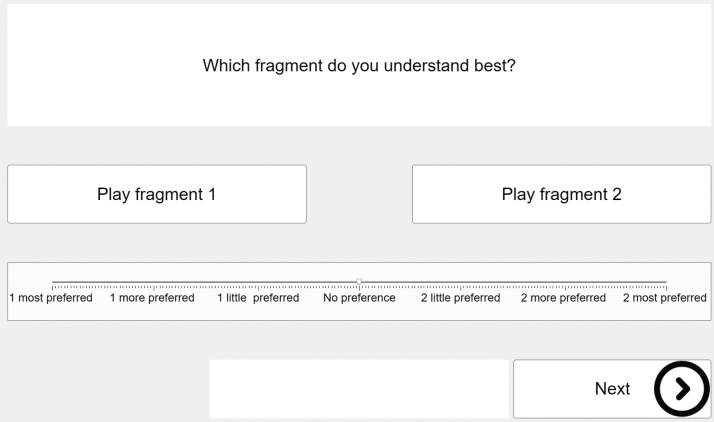
The graphical interface for the subjective ratings. Participants used the slider to indicate whether they preferred one of the two fragments.

To assess how study participants experienced the processed signals, the participants were asked to compare pairs of stimuli and to indicate their subjective preference (if any) for one of the two stimuli. Both stimuli in a pair contained the same sentence (one from the Matrix material) but were processed differently. The participants’ preference was based on three sound pleasantness outcomes, speech intelligibility, naturalness, and listening effort, which were extracted from the “general” section of the Spatial Audio Quality Inventory (Looi et al. 2011; Lindau et al. 2014). The pairs of stimuli were presented via a custom-made graphical user interface in a MATLAB environment (version R2023a), which the participants accessed on a touchscreen tablet (16T2, 15.6 inch; AOC, Taipei, Taiwan). Participants could listen to each fragment as many times as they wanted. They were asked to use a bipolar slider marked with Likert captions to indicate the magnitude of their subjective preference for either of the two fragments (Fig. 1). The final position of the slider was translated to a Likert score ranging from 0 to 100, with 50 representing no preference for either fragment.

The presented stimulus pairs were divided into measurement and control measurements. The measurements included the following pairs: clean speech versus reverberated speech; clean speech versus reverberated speech with DNN-WPE; clean speech versus reverberated speech with DNN-WPEPF; and reverberated speech with DNN-WPE versus reverberated speech with DNN-WPEPF. The control measurements were performed to analyze the effect of the algorithms on clean speech and were as follows: clean speech versus clean speech with DNN-WPE; clean speech versus clean speech with DNN-WPEPF; clean speech with DNN-WPE versus clean speech with DNN-WPEPF; and reverberated speech versus reverberated speech (Fig. 4). When the sentences contained reverberation (reverberated speech, reverberated speech with DNN-WPE, and reverberated speech with DNN-WPEPF), their previously determined RT60_50%_ was used during the subjective ratings.

All pairs of measurements and control measurements were tested using a randomized block design. In each block, participants assessed one of the subjective outcomes: speech intelligibility, naturalness, and listening effort. They performed a test and a retest of the subjective ratings, and the test-retest variability was assessed by calculating the intraclass correlation coefficient (ICC) (Koo & Li 2016) and its 95% confidence interval, following the ICC3 procedure described by Shrout and Fleiss (1979). The ICC3 was computed using the function *Intraclass_corr* in Python (version 3.10).

### Objective Assessment of the Effectiveness of the Dereverberation Algorithms

**Fig. 2.**
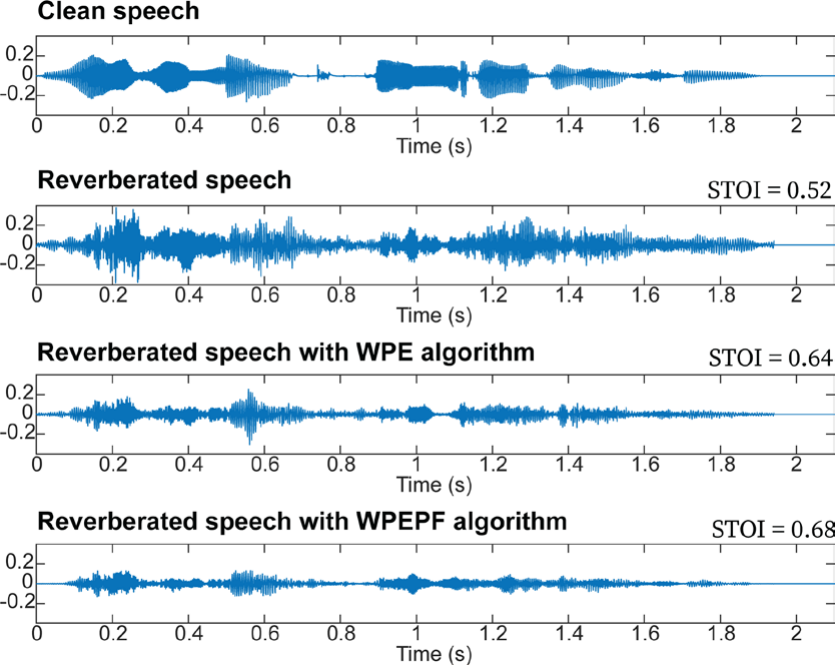
Raw speech signals for clean speech, reverberated speech, reverberated speech with the DNN-WPE algorithm, and reverberated speech with the DNN-WPEPF algorithm. The presented STOI scores are relative to clean speech (value of 1). DNN indicates deep neural network; DNN-WPE, DNN-based weighted-prediction error; DNN-WPEPF, weighted-prediction error and post-filter; STOI, short-time objective intelligibility.

Simulation-based calculations were performed to predict the effects of the algorithms with the Dutch/Flemish matrix materials, and to compare our results with the modeled outcomes of Lemercier et al. (2022, 2023). To estimate the perceived advantages of each algorithm, we calculated the short-time objective intelligibility (STOI) outcome according to the methods described by Taal et al. (2011a, 2011b). This measure indicates speech intelligibility based on calculations of the correlation coefficient between the temporal envelopes of the clean and degraded speech signals, yielding a value between 0 and 1. Because the temporal envelope is used in the speech processing of CIs, it is considered a predictor of speech intelligibility during in vivo CI use (Falk et al. 2015). The STOI was calculated separately for all 260 Matrix sentences and the average of all sentences per condition. The STOI was computed using an RT60 of 1 sec, which was the average RT60_50%_ among our study participants. Clean speech was used as the reference signal (value 1). Figure 2 presents an example of one sound fragment and its STOI for the different conditions.

### Statistics

Statistical analyses of the speech intelligibility output and subjective ratings were performed using SPSS version 29 for Windows (IBM Corp., Armonk, NY). To analyze the speech intelligibility results, a linear mixed model (LMM) was constructed (Eq. 1). As outcome parameter of this LMM, the percentage correct scores were transformed to rationalized arcsine units (RAU), as described by Studebaker (1985). With this approach, the high values (close to 100%) and low values (close to 0%) are transformed to create a more normalized dataset and reduce Type II errors. The LMM consisted of the RAU score as the outcome parameter, subject number as a random effect, and speech condition (i.e., with or without reverberation, and with or without postprocessing with a dereverberation algorithm) as a repeated fixed effect according to:


$$\begin{array}{lll} RAU\;score= & \;Intercept+Speech\;condition\; & +1|Subject\;number \end{array}$$ (1)

A second LMM analysis was performed to investigate the effect of RT60_50%_ on the speech intelligibility scores. The LMM included the RAU score as the outcome parameter, subject number as a random effect, and speech condition as a repeated fixed effect. However, only the speech conditions with reverberation were included in this model (reverberated speech and reverberated speech with either algorithm). In addition, RT60_50%_ and the interaction factor, Reverberated Condition × RT60_50%,_ were included (Eq. 2).


$$\begin{array}{l} \begin{array}{lll} RAU\;score= & \;Intercept+Reverberated\;condition+RT60_{50\%}\; & +\;Reverberated\;condition\times RT60_{50\%}+1|Subject\;number\; \end{array} \end{array}$$ (2)

To optimize both models, we compared the Bayesian information criteria and Akaike’s information criteria for six variance/covariance structures suitable for longitudinal data: scaled identity, compound symmetry, correlation compound symmetry, heterogeneous compound symmetry, unstructured, and unstructured correlation. On the basis of these results, the covariance matrix of both LMMs was set to “scaled identity.” The restricted maximum likelihood procedure was used during optimization. Post hoc pairwise comparisons were executed using Šidák correction for multiple comparisons.

The mean values of the test and retest of the pairs during the subjective ratings were statistically evaluated using a one-sample, two-tailed *t* test. These *t* tests were performed for every comparison with a null hypothesis of 50 (no preference). Bonferroni multiple-comparison correction was applied for each subjective outcome measure.

To provide insight into the differences for the objective measure STOI, we performed three paired one-sample, two-tailed *t* tests. The first *t* test compared the STOI of reverberated speech versus the STOI of DNN-WPE dereverberated speech, the second compared the STOI of reverberated speech versus the STOI of DNN-WPEPF dereverberated speech, and the last compared the STOI of DNN-WPE dereverberated speech with the STOI of DNN-WPEPF dereverberated speech. A Bonferroni multiple-comparison correction was applied to correct for the three tests.

## RESULTS

### Objective Assessment of the Effectiveness of the Dereverberation Algorithms

Figure 2 shows the waveforms of monaural speech signals, representing a single sentence without reverberation (clean speech), reverberated speech (RT60 = 1.0 sec), and speech that was dereverberated using each of the two algorithms. For all 260 matrix sentences, with RT60 of 1 sec, the mean STOI was 0.52 (SD = 0.031) for reverberated speech, 0.65 (SD = 0.034) for reverberated speech with DNN-WPE, and 0.68 (SD = 0.031) for reverberated speech with DNN-WPEPF, indicating that predicted speech intelligibility was improved by 12% with DNN-WPE, and by 16% with DNN-WPEPF. The differences in STOI between reverberated speech and reverberated speech with DNN-WPE or DNN-WPEPF, as well as between reverberated speech with DNN-WPE and reverberated speech with DNN-WPEPF, were statistically significant according to the performed *t* tests with Bonferroni correction (*p* < 0.001).

### Speech Intelligibility

**Fig. 3.**
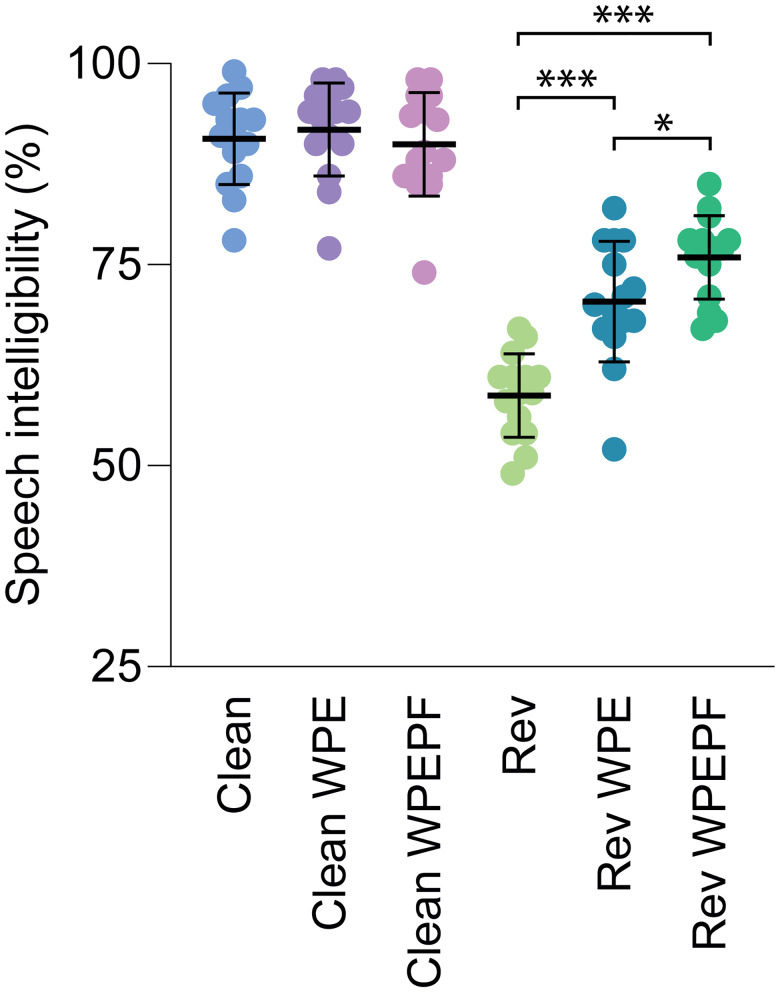
Speech intelligibility scores for six different conditions: clean speech (clean), clean speech with the DNN-WPE (clean WPE), clean speech with the DNN-WPEPF (clean WPEPF), reverberated speech (Rev), reverberated speech with the DNN-WPE (Rev WPE), and reverberated speech with the DNN-WPEPF algorithm (Rev WPEPF). *p < 0.05, ***p < 0.001. DNN indicates deep neural network; DNN-WPE, DNN-based weighted-prediction error; DNN-WPEPF, weighted-prediction error and post-filter.

Figure 3 presents the speech intelligibility scores, revealing an average improvement of 11% when reverberated speech was processed with DNN-WPE, and 17% when processed with DNN-WPEPF (Fig. 3). The first LMM with all speech conditions (which was performed with RAU scores instead of percentages correct) revealed a statistically significant main effect of “speech condition” (*F* = 113.4, *p* < 0.001). Post-hoc pairwise comparisons showed statistically significant benefits of both DNN-WPE (*p* < 0.001, df = 160) and DNN-WPEPF (*p* < 0.001, df = 160) over the reverberated condition. Moreover, the benefit of DNN-WPEPF was significantly greater than the benefit of DNN-WPE (*p* = 0.047, df = 160). On the other hand, neither algorithm significantly affected the intelligibility of clean speech, with differences of 1% (WPE: *p* = 0.842, df = 160, WPEPF: *p* = 0.995, df = 160).

The second LMM was used to analyze the effect of RT60_50%_ on the speech intelligibility scores (converted to RAU). Neither the main effect of RT60_50%_ nor the interaction between Condition × RT60_50%_ was statistically significant (RT60_50%_: *F* = 0.994, *p* = 0.337, interaction factor: *F* = 1.26, *p* = 0.290), meaning that RT60_50%_ did not significantly influence the benefit of either algorithm, at least up to an RT60_50%_ of 1.5 sec.

### Subjective Ratings

**Fig. 4.**
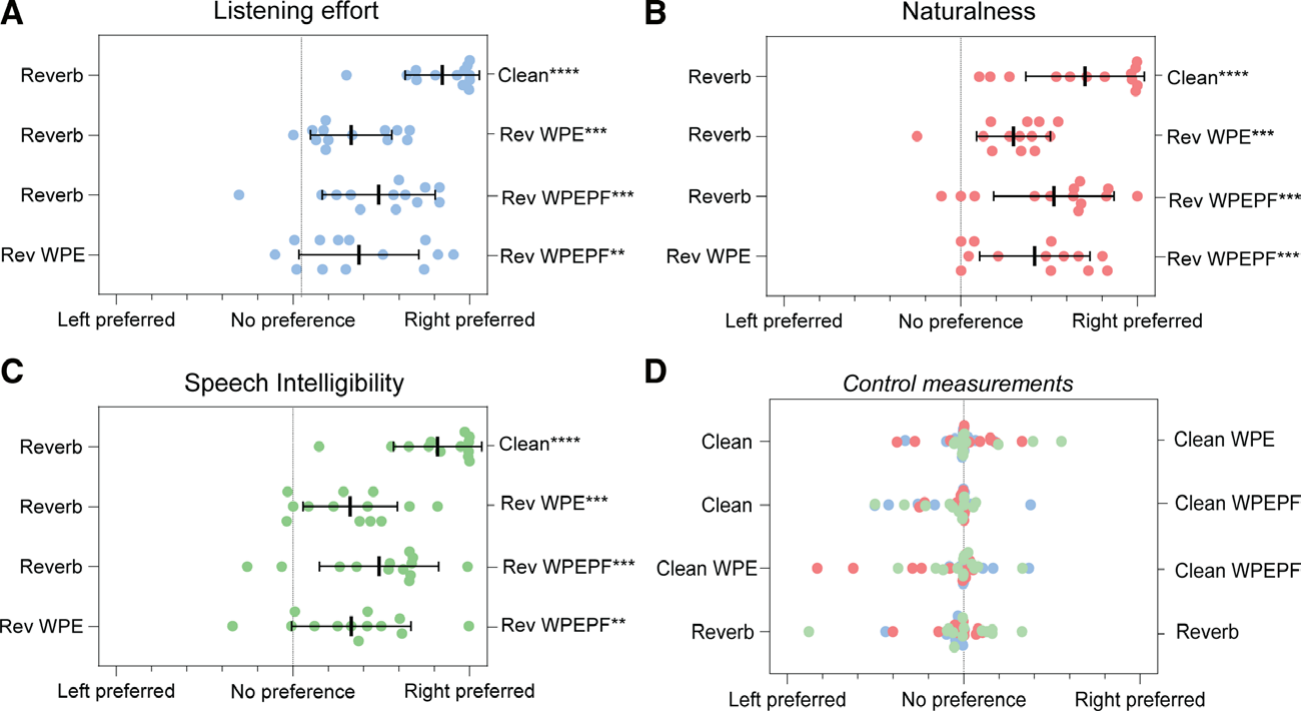
Subjective ratings for (A) listening effort, (B) naturalness, and (C) speech intelligibility are presented in scatter plots, showing the mean outcomes and SDs. For all three outcomes, participants indicated statistically significant preferences for clean speech over reverberated speech, for reverberated speech processed with either algorithm compared with unprocessed reverberated speech, and for speech processed using the DNN-WPEPF algorithm over the DNN-WPE algorithm. **p < 0.01, ***p < 0.001, ****p < 0.0001. (D) Scatterplots of the control measurements for all three outcomes, showing no statistically significant differences. Clean WPE, clean speech with the weighted-prediction error algorithm; Clean WPEPF, clean speech with the weighted-prediction error and post-filter algorithm; Reverb WPE, reverberated speech with the weighted-prediction error algorithm; Reverb WPEPF, reverberated speech with the weighted-prediction error and post-filter algorithm.

Figure 4 presents the results from the subjective ratings of listening effort, naturalness, and speech intelligibility. All three outcomes showed similar results, which aligned with the speech intelligibility scores. Participants significantly preferred clean speech over reverberated speech (Table 2, Figs. 4A–C). In terms of all three subjective outcomes, speech dereverberated with either algorithm was significantly preferred over reverberated speech (Table 2, Figs. 4A–C). Moreover, speech dereverberated with DNN-WPEPF was significantly preferred over speech dereverberated with DNN-WPE (Table 2, Figs. 4A–C).

**TABLE 2.** Participants’ subjective ratings of naturalness, speech intelligibility, and listening effort

	Naturalness		Speech Intelligibility		Listening Effort	
	*t* Value	*p*	*t* Value	*p*	*t* Value	*p*
Reverberation vs. clean speech	7.55	<0.0001	11.84	<0.0001	14.49	<0.0001
Reverberation vs. Reverb WPE	5.17	0.0002	4.38	0.0009	5.15	0.0002
Reverberation vs. Reverb WPEPF	5.58	0.0001	5.22	0.0002	5.45	0.0001
Reverb WPE vs. Reverb WPEPF	4.85	0.0004	3.54	0.0041	3.96	0.0019
Clean speech vs. Clean WPE	0.32	0.75	1.53	0.15	0.63	0.54
Clean speech vs. Clean WPEPF	1.65	0.12	1.83	0.09	1.15	0.27
Clean WPE vs. Clean WPEPF	1.91	0.079	0.24	0.81	1.35	0.20
Reverberation vs. reverberation	0.85	0.41	0.29	0.78	1.78	0.10

Outcomes were analyzed by *t* test.

Clean WPE, clean speech with the weighted-prediction error algorithm; Clean WPEPF, clean speech with the weighted-prediction error and post-filter algorithm; Reverb WPE, reverberated speech with the weighted-prediction error algorithm; Reverb WPEPF, reverberated speech with the weighted-prediction error and post-filter algorithm.

In addition, pairwise controls were performed by assessing whether the algorithms affected clean speech. None of the control measurements showed a statistically significant difference (Table 2); therefore, the results for the three subjective outcomes are combined in Figure 4D. To assess the accuracy of our method, we also compared reverberated speech to reverberated speech, and participants indicated no preference, as expected. The test-retest variability was represented by an ICC3 of 0.80, indicating “good” intra-rater reliability, with a 95% confidence interval of 0.61 to 0.94, corresponding to a range from “moderate” to “excellent” reliability (Koo & Li 2016).

## DISCUSSION

This study evaluated two versions of a novel artificial-intelligence-based algorithm that reduces reverberation in speech (Lemercier et al. 2022, 2023). The DNN-WPE version of the algorithm targeted early reverberation, whereas the DNN-WPEPF version included an additional post-filtering step to reduce early, and late reverberation, specifically for use in CIs. Both algorithms yielded statistically significant improvements in speech intelligibility—DNN-WPE by 11%, and DNN-WPEPF by 17%. When applied to clean speech (without reverberation), the algorithms did not significantly affect speech understanding scores, indicating that they can be active even in the absence of reverberation. In addition to extending previous acoustic assessments of the dereverberation approaches by testing outcomes for CI listeners, another key advancement of this study was to include the participants’ subjective ratings of listening effort, speech intelligibility, and naturalness. These subjective ratings demonstrated a significant preference for dereverberated speech over reverberated speech not processed with the algorithms. Notably, CI users preferred speech processed by the DNN-WPEPF algorithm compared with the DNN-WPE algorithm, confirming the greater effectiveness of reducing early and late reverberations rather than only early reverberations (Lemercier et al. 2022, 2023).

To compare our clinical outcomes with the objective effectiveness of the algorithms as described in Lemercier et al. (2022), STOI values were calculated for the complete speech corpus of the Dutch/Flemish Matrix test, using an RT60 of 1 sec. We found STOI improvements of 12% with DNN-WPE, and 16% with DNN-WPEPF. These numbers closely corresponded to the improvements of the speech intelligibility test outcomes obtained in our group of CI users (11% for DNN-WPE, and 17% for DNN-WPEPF). These results support that STOI can be a predictor of CI speech intelligibility for the materials and processing conditions evaluated in this study. Lemercier et al. reported 16% objective effectiveness of the DNN-WPE algorithm, which was also based on the STOI. The use of a different speech corpus might explain the discrepancy between STOI values. In our study, the Dutch/Flemish Matrix material was used, whereas Lemercier et al. calculated STOI values using the WHAMR dataset, which is based on American English speech of the Wall Street Journal (Garofolo et al. 1993; Maciejewski et al. 2020).

Although simulations and objective measures provide insight into which algorithms may work for CI users, they cannot indicate how the processed speech is actually perceived. For instance, an algorithm may improve speech intelligibility in terms of percentage correct scores, but still produce distorted speech that sounds unpleasant, potentially leading CI users to avoid using that setting. Several dereverberation methods have been assessed among CI users, including the ideal reverberant masking approach (Kokkinakis et al. 2011; Hazrati & Loizou 2013; Shahidi et al. 2022), blind binary masking approach (Hazrati et al. 2013), and spectral subtraction strategy (Kokkinakis et al. 2015). Both tested masking approaches increased speech intelligibility by between 30% and 40% for CI users (Kokkinakis et al. 2011; Hazrati & Loizou 2013; Hazrati et al. 2013), indicating benefits substantially larger than those reported in our present study. However, both masking methods relied on a priori knowledge of the non-reverberant signal. By contrast, the presently tested DNN-WPE and DNN-WPEPF algorithms do not require a priori knowledge; they are trained on 101 speakers and tested on different speech materials and languages that have not been seen during training. Also, the RIRs during training and testing do not overlap.

The spectral subtraction strategy tested by Kokkinakis et al. (2015) yielded 15% improvement in speech intelligibility for an RT60 of 0.3 sec, and 25% improvement for an RT60 of 1.0 sec. Gaultier and Goehring (2024) described an improvement in speech recognition threshold of 8.5 dB for a neural network-based dereverberation algorithm with access to six microphones and an improvement of 3.8 dB when using a two-microphone input. This two-microphone input is comparable to our setup with one microphone signal from both sides of the head. Contrary to the studies of Kokkinakis et al. and Gaultier and Goehring, an advantage of the present study was the inclusion of subjective ratings, which demonstrated that the DNN-WPE and DNN-WPEPF algorithms were subjectively preferred over unprocessed reverberated speech, in terms of speech intelligibility, naturalness, and listening effort.

An aspect that warrants closer examination is the training paradigm of the DNN-WPE and DNN-WPEPF algorithms. Both algorithms were trained on RT60 values ranging from 0.4 to 1 sec. However, we also observed increased speech intelligibility for participants with an RT60_50%_ of >1 sec, and these participants did not receive reduced benefits of dereverberated speech with the algorithms. This suggests that the algorithms also work for higher reverberation times, at least up to 1.5 sec.

Another limitation of our study is that both the training and test sets were generated using the same reverberation procedure, with variations only in RT60 and room dimensions. However, the room dimensions used during the tests performed in this study were different than those during training, and the RT60s were extended up to 1.5 sec in contrast with the range of 0.4 to 1 sec during training. We decided to maintain the same source-listener distance during testing (which was different from training) to limit the number of test conditions and maintain experimental feasibility. For potential application in CI speech processors, it will be important to determine whether the algorithms still provide benefits when applied in rooms with different dimensions and characteristics than those used during the tests in this study. Future studies should also take a varying DRR and varying source-listener distances into account because these factors play an important role in the speech intelligibility of CI users (Kressner et al. 2018; Eurich et al. 2019; Badajoz-Davila et al. 2020). The benefit of the algorithms will likely be smaller in typical room environments encountered in daily life than reported here, because we measured at the participants' RT60_50%_, which is already a challenging condition (median RT60_50%_ of 1 sec, many typical room environments would have lower RT60s).

Last, as mentioned, the algorithms were trained using American English speech material. Our present results indicate that the Dutch language does not seem to present a limitation regarding the efficiency of the algorithms. However, Western languages are generally similarly structured in contrast with tonal languages, such as Mandarin Chinese (Baker 2003). This suggests that Western languages may be used interchangeably with such speech filtering models, while tonal languages may pose a greater challenge for these algorithms.

Future research should focus on the effectiveness and implementation of the algorithms in real-life situations. These are situations where reverberation occurs in conjunction with other noise sources, like competing talkers. Hazrati and Loizou (2012) have reported that the combination of noise sources (including reverberation) often occurs in daily real-life situations. The algorithms have not been trained or tested in situations involving reverberation combined with other noise, and it will be interesting to determine whether they still increase speech intelligibility in such settings, as shown in Gaultier and Goehring (2024). Another key aspect to analyze is how the studied dereverberation algorithms interact with existing noise reduction algorithms.

It is also important to analyze the effects of the initialization period and algorithmic latency on the experience of CI users when available in daily life. The algorithm needs at most 2 sec of initialization time, when the algorithm is activated from silence (start-up phase), before it performs optimally (unpublished observations). The initialization period is significantly lower when transitioning between environments, as the changes are less pronounced compared with the start-up phase. It is known that the algorithm has more difficulty when sudden or fast movements or changes in environments occur (head movement of the listener is more disruptive for the effectiveness of the algorithms than a source slowly walking through the room). Therefore, it is important to test the algorithms in real-life situations with CI users.

Another aspect is the induced latency when the algorithm is activated, which is 40 msec at present (Lemercier et al. 2023). The processing latency is hardware-dependent, and as of now, we do not know what the typical processing latency on current commercial CI processors would be for the given algorithm, especially because the Python code would first need to be translated to lower-level code, such as, for example, C/C++. However, latency is possibly not a limiting factor. Although typical figures of tolerated latencies in HAs are on the order of 20 msec, in CI users, the tolerated latency is dependent on residual hearing in the contralateral ear. When no residual hearing is left, self-speech situations are absent because CI users solely rely on their CI and cannot hear their own voice without the implant. To the extent of our knowledge, the tolerated latency of those CI users is mainly dictated by the audio-visual synchronicity, which is 100 to 200 msec in typical humans (Hay-McCutcheon et al. 2009). Therefore, the algorithmic latency of 40 msec is possibly not a limiting factor, especially for CI users without residual hearing.

In conclusion, this study revealed that the DNN-WPE and DNN-WPEPF dereverberation algorithms had benefits for CI users, in terms of speech intelligibility and subjective ratings. These algorithms did not affect clean speech, implying that they can be switched on in quiet situations without background noise. Further research is needed to confirm the effects of these algorithms in real time when implemented in the CI processor, and to test their use under various realistic listening conditions.

## ACKNOWLEDGMENTS

The authors thank the study participants for their time and dedication, and Advanced Bionics and Hamburg University for their technical support.
